# Supplementary material for: A cross-sectional study of physicians on fluoride-related beliefs and practices, and experiences with fluoride-hesitant caregivers
Source: PLoS One. 2024 Jul 19;19(7):e0307085. doi: 10.1371/journal.pone.0307085 (PMC11259263; doi:10.1371/journal.pone.0307085)
Supplement: S1 File — (PDF) [file pone.0307085.s002.pdf]

Codebook

Data Dictionary Codebook

10/11/2021 10:53am

Collapse all instruments

#	Variable / Field Name	Field Label *Field Note*	Field Attributes (Field Type, Validation, Choices, Calculations, etc.)
Instrument: **Provider Survey on Fluoride** (provider_survey_on_fluoride) Enabled as survey Collapse			
1	participant_id	Participant ID	text

2	consent	UNIVERSITY OF WASHINGTON CONSENT FORM Title of Study: Medical Provider Fluoride-Prescribing Patterns Principal Investigator: Dr. Donald Chi, DDS, PhD, 206-616-4332 Study Coordinator: Dr. Tiffany Bass, DDS, 360-338-5682 CONSENT TO TAKE PART IN A RESEARCH STUDY This online consent form is part of an informed consent process for a research study. It will give you information to help you decide whether to be in the study or not. Please read the form carefully. It is your choice to take part or not. You may ask questions if anything on this form is not clear. If you decide to take part, the instructions at the end of the document will tell you what to do next. Who is conducting this research study? You are being asked to take part in research conducted by Dr. Donald Chi, a pediatric dentist and professor in the University of Washington School of Dentistry along with Dr. Tiffany Bass, a dentist and graduate student at the University of Washington School of Public Health. What is this research study about? We are conducting this study to learn more about medical providers' knowledge of fluoride and fluoride-prescribing patterns. This information will be used to develop continuing education courses about fluoride for medical providers. What will I be asked to do if I take part? The online survey will take approximately 5 minutes to complete. The survey will have questions about your background and your type of practice, questions about your knowledge and understanding of fluoride and questions about when you prescribe fluoride. Your participation is voluntary. You can refuse to participate or withdraw your participation at any time with no penalty or loss of benefits to which you are otherwise entitled. What are the risks and/or discomforts I might experience if I take part in the study? While we do not anticipate any risks from taking part in the study, it is possible some questions may cause slight discomfort. Breach of confidentiality is a risk of harm, but there is a data security plan in place to minimize this risk. There is a low likelihood of these risks. Are there any benefits to me if I choose to take part in this study? There are no direct benefits to you for taking part in this research. We hope that the information you provide will help researchers develop fluoride education interventions for medical providers. Will I be paid to take part in this study? There is no payment for your participation in the study. How will information about me be kept private or confidential? All efforts will be made to keep your data confidential. The link between any personal identifiers and the research data will be destroyed after the records retention period required by state and/or federal law. No one outside of the research team will have access to identifiable data. Who is funding this research study? This study is not funded. Who can I call if I have questions? If you have questions about this study, or feel you have been harmed by participating, you can contact the Principal Investigator: Dr. Donald Chi at the University of Washington School of Dentistry, at (206) 616-4332 or email dchi@uw.edu. If you have questions about your rights as a research participant, you can call the University of Washington IRB Human Subject Division at (206) 543-0098 or call collect at (206) 221-5940 or email hsdinfo@uw.edu. Please print out this consent form if you would like a copy of it for your files. If you do not want to take part in the research, close this webpage. If you do want to part in this research, follow the directions below: By beginning this research, I acknowledge that I am 18 years of age or older and have read and understand the information given to me. I agree to take part in the research, with the knowledge that I am free to withdraw my participation in the research without penalty. Click "Next Page" to confirm informed consent and begin the survey.	descriptive
3	screener_2	Section Header:<br>Are you a family medicine physician or pediatrician?	yesno, Required<br>1 Yes<br>0 No

4	screener_no_2<br>Show the field ONLY if:<br>[screener_2] = '0'	Thank you for your interest in participating in our survey. Unfortunately, you do not meet the eligibility criteria.	descriptive
5	screener<br>Show the field ONLY if:<br>[screener_2] = '1'	Does your practice treat child patients (younger than age 18 years)?	yesno, Required<br>1 Yes<br>0 No
6	screener_no<br>Show the field ONLY if:<br>[screener] = '0'	Thank you for your interest in participating in our survey. Unfortunately, you do not meet the eligibility criteria.	descriptive
7	eff_1<br>Show the field ONLY if:<br>[screener] = '1' and [screener _2] = '1'	Section Header: *Effectiveness of Fluoride*<br>In general, how effective do you believe fluoride is in preventing cavities?	radio<br>0 Very effective<br>1 Effective<br>2 Somewhat effective<br>3 Not effective
8	eff_2<br>Show the field ONLY if:<br>[screener] = '1' and [screener _2] = '1'	How effective do you believe prescription fluoride supplements are in preventing cavities?	radio<br>0 Very effective<br>1 Effective<br>2 Somewhat effective<br>3 Not effective
9	eff_3<br>Show the field ONLY if:<br>[screener] = '1' and [screener _2] = '1'	How effective do you believe topical fluoride (e.g. varnish) is in preventing cavities?	radio<br>0 Very effective<br>1 Effective<br>2 Somewhat effective<br>3 Not effective
10	eff_4<br>Show the field ONLY if:<br>[screener] = '1' and [screener _2] = '1'	How effective do you believe over-the-counter fluoride toothpastes are in preventing cavities?	radio<br>0 Very effective<br>1 Effective<br>2 Somewhat effective<br>3 Not effective
11	eff_5<br>Show the field ONLY if:<br>[screener] = '1' and [screener _2] = '1'	How effective do you believe fluoridated water is in preventing cavities?	radio<br>0 Very effective<br>1 Effective<br>2 Somewhat effective<br>3 Not effective
12	curr_1<br>Show the field ONLY if:<br>[screener] = '1' and [screener _2] = '1'	Section Header: *Current Fluoride-Related Practices*<br>Do you currently prescribe fluoride supplements (like tablets, drops) to your patients?	yesno<br>1 Yes<br>0 No
13	curr_1yes<br>Show the field ONLY if:<br>[curr_1] = '1'	At what age do you start prescribing fluoride supplements?	radio<br>0 Birth to 6 months<br>1 6 months to 3 years<br>2 3 years to 6 years<br>3 6 years and older

14	curr_1no<br>Show the field ONLY if:<br>[curr_1] = '0'	What are the reasons you do not currently prescribe fluoride supplements? (Choose all that apply)	checkbox<br>0 | curr_1no___0 | My patients are at low risk for cavities<br>1 | curr_1no___1 | My patients live in an area with fluoridated water<br>2 | curr_1no___2 | There is no longer a need for fluoride supplements<br>3 | curr_1no___3 | There are too many barriers for my patients to fill a fluoride prescription<br>4 | curr_1no___4 | Many of my patients are hesitant about prescription fluoride supplements<br>5 | curr_1no___5 | Other
15	curr_1no_other<br>Show the field ONLY if:<br>[curr_1no(5)] = '1'	You chose "other" to reasons for not currently prescribing fluoride supplements. Please specify.	notes
16	curr_2<br>Show the field ONLY if:<br>[screener] = '1' and [screener_2] = '1'	Do you currently offer topical fluoride (e.g. varnish) application to your patients?	yesno<br>1 | Yes<br>0 | No
17	curr_2yes<br>Show the field ONLY if:<br>[curr_2] = '1'	At what age do you start applying topical fluoride?	radio<br>0 | Birth to 6 months<br>1 | 6 months to 3 years<br>2 | 3 years to 6 years<br>3 | 6 years and older
18	curr_2no<br>Show the field ONLY if:<br>[curr_2] = '0'	What are the reasons you do not currently offer topical fluoride application? (Choose all that apply)	checkbox<br>0 | curr_2no___0 | My patients are at low risk for cavities<br>1 | curr_2no___1 | My patients live in an area with fluoridated water<br>2 | curr_2no___2 | There is no longer a need for topical fluoride<br>3 | curr_2no___3 | Many of my patients are hesitant about topical fluoride<br>4 | curr_2no___4 | Other
19	curr_2no_other<br>Show the field ONLY if:<br>[curr_2no(4)] = '1'	You chose "other" to reasons for not currently offering topical fluoride application. Please specify.	notes
20	curr_3<br>Show the field ONLY if:<br>[screener] = '1' and [screener_2] = '1'	Do you currently recommend the use of over-the-counter fluoride toothpaste to your patients?	yesno<br>1 | Yes<br>0 | No
21	curr_3yes<br>Show the field ONLY if:<br>[curr_3] = '1'	At what age do you start recommending the use of over-the-counter fluoride toothpaste?	radio<br>0 | Birth to 6 months<br>1 | 6 months to 3 years<br>2 | 3 years to 6 years<br>3 | 6 years and older

22	curr_3no<br><br>Show the field ONLY if:<br>[curr_3] = '0'	What are the reasons you do not recommend the use of over-the-counter fluoride toothpaste? (Choose all that apply)	checkbox<br>0 curr_3no___0 My patients are at low risk for cavities<br>1 curr_3no___1 My patients live in an area with fluoridated water<br>2 curr_3no___2 There is no longer a need for over-the-counter fluoride toothpaste<br>3 curr_3no___3 Many of my patients are hesitant about over-the-counter fluoride toothpaste<br>4 curr_3no___4 Other
23	curr_3no_other<br><br>Show the field ONLY if:<br>[curr_3no(4)] = '1'	You chose "other" to reasons for not recommending the use of over-the-counter fluoride toothpaste. Please specify.	notes
24	curr_4<br><br>Show the field ONLY if:<br>[screener] = '1' and [screener _2] = '1'	How strongly do you support water fluoridation?	radio<br>0 Strongly support<br>1 Somewhat support<br>2 Somewhat oppose<br>3 Strongly oppose
25	curr_5<br><br>Show the field ONLY if:<br>[screener] = '1' and [screener _2] = '1'	How often do parents or patients ask you for advice about fluoride?	radio<br>0 Never<br>1 Sometimes<br>2 Often<br>3 Always
26	curr_5ans<br><br>Show the field ONLY if:<br>[curr_5] = '1' or [curr_5] = '2' o r [curr_5] = '3'	What issues do your patients or parents of patients raise about fluoride? (Choose all that apply)	checkbox<br>0 curr_5ans___0 Conflicting advice from another healthcare provider<br>1 curr_5ans___1 Side effects<br>2 curr_5ans___2 Unsure of whether their child needs it<br>3 curr_5ans___3 Cost<br>4 curr_5ans___4 Patients never ask for advice about fluoride<br>5 curr_5ans___5 Other
27	curr_5other<br><br>Show the field ONLY if:<br>[curr_5ans(5)] = '1'	You chose "other" to issues patients or parents of patients raise about fluoride. Please specify.	notes
28	flh_1<br><br>Show the field ONLY if:<br>[screener] = '1' and [screener _2] = '1'	Section Header: *Fluoride Hesitancy The following items are related to fluoride hesitancy, which is similar to vaccine hesitancy.*<br>How problematic is fluoride hesitancy among patients in your practice?	radio<br>0 A big problem<br>1 A medium-sized problem<br>2 A small problem<br>3 Not a problem at all
29	flh_2<br><br>Show the field ONLY if:<br>[screener] = '1' and [screener _2] = '1'	Over time, fluoride hesitancy among patients in your practice has	radio<br>0 Gotten worse (more parents are refusing)<br>1 Stayed about the same<br>2 Gotten better (fewer parents are refusing)<br>3 I don't know

30	flh_3<br>Show the field ONLY if:<br>[screener] = '1' and [screener_2] = '1'	When you encounter a parent who is hesitant about fluoride, how comfortable are you talking to the parent about changing their mind about fluoride?	radio<br>0 Extremely comfortable<br>1 Somewhat comfortable<br>2 Somewhat uncomfortable<br>3 Extremely uncomfortable
31	flh_4<br>Show the field ONLY if:<br>[screener] = '1' and [screener_2] = '1'	What barriers do you face in incorporating oral health activities, like prescribing fluoride, into practice? (Choose all that apply)	checkbox<br>0 flh_4___0 Lack of knowledge<br>1 flh_4___1 Need to address other more important issues during visits<br>2 flh_4___2 Lack of time<br>3 flh_4___3 Lack of parent or patient interest<br>4 flh_4___4 Belief that oral health activities should be performed by dentists<br>5 flh_4___5 Lack of dentists in the area for referral<br>6 flh_4___6 Lack of reimbursement<br>7 flh_4___7 Other
32	flh_4other<br>Show the field ONLY if:<br>[flh_4(7)] = '1'	You chose "other" to barriers in incorporating oral health activities, like prescribing fluoride, into practice. Please specify.	notes
33	flh_5<br>Show the field ONLY if:<br>[screener] = '1' and [screener_2] = '1'	Please add any comments or clarifications.	notes
34	vacc_1<br>Show the field ONLY if:<br>[screener] = '1' and [screener_2] = '1'	Section Header: *Vaccine Hesitancy*<br>How often do parents or patients ask you for advice about vaccines?	radio<br>0 Never<br>1 Sometimes<br>2 Often<br>3 Always
35	vacc_1ans<br>Show the field ONLY if:<br>[vacc_1] = '1' or [vacc_1] = '2' or [vacc_1] = '3'	What issues do your parents or patients raise about vaccines? (Choose all that apply)	checkbox<br>0 vacc_1ans___0 Conflicting advice from another healthcare provider<br>1 vacc_1ans___1 Side effects<br>2 vacc_1ans___2 Unsure of whether their child needs it<br>3 vacc_1ans___3 Cost<br>4 vacc_1ans___4 Other
36	vacc_1other<br>Show the field ONLY if:<br>[vacc_1ans(4)] = '1'	You chose "other" to issues your parents or patients raise about vaccines. Please specify.	notes
37	vacc_2<br>Show the field ONLY if:<br>[screener] = '1' and [screener_2] = '1'	How problematic is vaccine hesitancy among patients in your practice?	radio<br>0 A big problem<br>1 A medium-sized problem<br>2 A small problem<br>3 Not a problem at all
38	vacc_3<br>Show the field ONLY if:<br>[screener] = '1' and [screener_2] = '1'	Over time, vaccine hesitancy among patients in your practice has	radio<br>0 Gotten worse (more parents are refusing)<br>1 Stayed about the same<br>2 Gotten better (fewer parents are refusing)<br>3 I don't know

39	vacc_4<br>Show the field ONLY if: [screener] = '1' and [screener _2] = '1'	When you encounter a parent who is hesitant about vaccines, how comfortable are you talking to the parent about changing their mind about vaccines?	radio<br>0 Extremely comfortable<br>1 Somewhat comfortable<br>2 Somewhat uncomfortable<br>3 Extremely uncomfortable
40	age<br>Show the field ONLY if: [screener] = '1' and [screener _2] = '1'	Section Header: *Demographics*<br>How old are you?<br>*Please indicate in years.*	text
41	sex<br>Show the field ONLY if: [screener] = '1' and [screener _2] = '1'	What is your gender?	radio<br>0 Woman/female<br>1 Man/male<br>2 Non-binary/third gender<br>3 Prefer to self-describe
42	sex_desc<br>Show the field ONLY if: [sex] = '3'	I self-describe as	notes
43	race<br>Show the field ONLY if: [screener] = '1' and [screener _2] = '1'	Please indicate your race.	radio<br>0 American Indian or Alaska Native<br>1 Asian<br>2 Black or African American<br>3 Native Hawaiian or other Pacific Islander<br>4 White<br>5 Other
44	race_other<br>Show the field ONLY if: [race] = '5'	You chose "other" for race. Please specify.	notes
45	ethnicity<br>Show the field ONLY if: [screener] = '1' and [screener _2] = '1'	Do you consider yourself to be Hispanic, Latino, or of Spanish origin?	yesno<br>1 Yes<br>0 No
46	providertype<br>Show the field ONLY if: [screener] = '1' and [screener _2] = '1'	Please indicate your provider type.	radio<br>0 Physician (family practice)<br>1 Physician (pediatrics)<br>2 Nurse practitioner (family practice)<br>3 Nurse practitioner (pediatrics)<br>4 Physician assistant (family practice)<br>5 Physician assistant (pediatrics)<br>6 Other
47	providertype_other<br>Show the field ONLY if: [providertype] = '6'	You chose "other" for provider type. Please specify.	notes
48	years<br>Show the field ONLY if: [screener] = '1' and [screener _2] = '1'	How many years have you been in practice since completing your residency/training?	radio<br>0 0-5 years<br>1 6-10 years<br>2 11-20 years<br>3 21-30 years<br>4 31+ years

49	state<br>Show the field ONLY if:<br>[screener] = '1' and [screener_2] = '1'	In which state do you practice?	text, Identifier
50	clinicset<br>Show the field ONLY if:<br>[screener] = '1' and [screener_2] = '1'	Which of the following best describes your main clinical practice setting?	radio<br>0 University or hospital<br>1 Community health center or public health clinic<br>2 Private practice<br>3 Military clinic<br>4 Other
51	clinicset_other<br>Show the field ONLY if:<br>[clinicset] = '4'	You chose "other" for your main clinical practice setting. Please specify.	notes
52	medicaid<br>Show the field ONLY if:<br>[screener] = '1' and [screener_2] = '1'	What % of patients in your practice are insured by Medicaid?	radio<br>0 0%<br>1 1-25%<br>2 26-50%<br>3 51-75%<br>4 more than 75%
53	tap<br>Show the field ONLY if:<br>[screener] = '1' and [screener_2] = '1'	Do you currently practice in an area where the tap water (drinking water) is fluoridated?	radio<br>1 Yes<br>0 No<br>2 I'm not sure
54	cont	Would you be interested in a Continuing Education course about fluoride?	yesno<br>1 Yes<br>0 No
55	futres	Would you be interested in participating in future research studies?	yesno<br>1 Yes<br>0 No
56	email<br>Show the field ONLY if:<br>[cont] = '1' or [futres] = '1'	Please indicate your email address.	notes, Identifier
57	addcomm<br>Show the field ONLY if:<br>[screener] = '1' and [screener_2] = '1'	Please provide any additional comments about survey items or things we may have missed.	notes
58	provider_survey_on_fluoride_complete	Section Header: *Form Status*<br>Complete?	dropdown<br>0 Incomplete<br>1 Unverified<br>2 Complete
