# Supplementary material for: A cross-sectional study of physicians on fluoride-related beliefs and practices, and experiences with fluoride-hesitant caregivers
Source: PLoS One. 2024 Jul 19;19(7):e0307085. doi: 10.1371/journal.pone.0307085 (PMC11259263; doi:10.1371/journal.pone.0307085)
Supplement: Attachment — Submitted filename: PONE-D-24-16749_reviewer.docx [file pone.0307085.s003.docx]

I thank the editor for providing this opportunity to participate in the manuscript review

As a reviewer, I find several strengths and areas for improvement in the study on fluoride-related beliefs and practices among pediatric and family medicine providers.

Strengths:

Clear Objectives and Findings Presentation: The study clearly outlines its objectives and presents its findings in a structured manner, making it easy for readers to understand the research aims and outcomes.

Relevant and Timely Topic: With increasing concerns about fluoride hesitancy among caregivers, especially mirroring vaccine hesitancy, the study addresses a pertinent issue in pediatric and family medicine practice.

Insightful Comparison between Specialties: The comparison between pediatric and family medicine providers provides valuable insights into potential differences in knowledge, beliefs, and practices regarding fluoride recommendations.

Identification of Barriers: The study effectively identifies common barriers to incorporating oral health activities into medical practice, particularly the lack of time, which is a practical concern for many healthcare providers.

Practical Implications: By highlighting the need to improve medical provider awareness about the benefits of fluoride and addressing fluoride hesitancy among caregivers, the study offers practical implications for enhancing preventive dental care in clinical settings.

Areas for Improvement:

Sampling Limitations: The use of convenience sampling in two states and the predominantly white physician sample limit the generalizability of the findings. Future studies should strive for more diverse and representative samples to ensure broader applicability of the results, especially considering the disproportionate impact of tooth decay on minority children.

Quantitative Data Analysis: While the study provides valuable quantitative data on providers' beliefs and practices, it would benefit from more robust statistical analyses to strengthen the validity and reliability of the findings.

Qualitative Insights: Incorporating qualitative methods, such as interviews or focus groups, could provide deeper insights into the reasons behind providers' beliefs, practices, and perceived barriers, enriching the understanding of fluoride-related attitudes in clinical practice.

Recommendation Strategies: While the study mentions the need to explore strategies for addressing fluoride hesitancy, it could elaborate further on specific intervention approaches or educational programs that could be implemented in medical settings to improve fluoride-related practices.

Addressing these areas for improvement could enhance the rigor and comprehensiveness of the study, thereby strengthening its contribution to the literature on preventive dental care in pediatric and family medicine.

Best Regards
